# Development of Optimized *Origanum vulgare* L. Essential Oil-Loaded Chitosan/Gum Arabic Nanocapsules by Complex Coacervation

**DOI:** 10.1021/acsomega.5c09153

**Published:** 2025-10-15

**Authors:** Sonálle C. A. Andrade, Ana F. C. Uchôa, Allessya L. D. Formiga, Anny L. M. R. Cardoso, Nereide S. S. Magalhães, Rodrigo O. França, Sócrates G. dos Santos, Marciane Magnani, Francisco H. Xavier-Junior, Thayza C. M. Stamford

**Affiliations:** † 28116 Federal University of Pernambuco, Avenida Professor Morais Rego, 1235, Cidade Universitária, 50670-901 Recife, Pernambuco, Brazil; ‡ Postgraduate Program in Bioactive Natural and Synthetic Products, 28097 UFPB, Campus I-Castelo Branco III, 58051-900 João Pessoa, Paraíba, Brazil; § Laboratory of Pharmaceutical Biotechnology (BioTecFarm), Department of Pharmaceutical Sciences, Center of Health Sciences, Federal University of Paraíba (UFPB), Campus I-Castelo Branco III, 58051-900 João Pessoa, Paraíba, Brazil; ∥ Keizo-Asami Institute (iLIKA), Federal University of Pernambuco (UFPE), 50670-901 Recife, Pernambuco, Brazil; ⊥ Faculty of Health Sciences, University of Manitoba, D226H-780 Bannatyne Avenue, R3E 0W2 Winnipeg, Manitoba, Canada; # Technology Center, Federal University of Paraíba (UFPB), Campus I-Castelo Branco III, 58051-900 João Pessoa, Paraíba, Brazil

## Abstract

Oregano essential oil (OEO), rich in carvacrol and thymol, has bioactive properties but is prone to degradation due to its volatile nature. Nanoencapsulation by complex coacervation, using chitosan (CHI) and gum arabic acid (GA), emerges as an alternative to increase its stability in food matrices. This study investigated the influence of the CHI/GA mass ratio on the formation of OEO-containing nanocapsules and quantified the encapsulated carvacrol using validated GC–MS. A Box–Behnken experimental design optimized the OEO concentration, CHI/GA ratio, and amount of Tween 80. Physicochemical properties such as the diameter, PdI, and zeta potential were evaluated. Morphology was analyzed by SEM and AFM, and thermal stability by TGA and DSC. Stability was monitored for 120 days at 4 °C, 25 °C, and 40 °C. The optimized formulation (470 mg of OEO, 659 mg of CHI/GA 1:5, and 13 mg of Tween) resulted in nanocapsules with a diameter of 323 ± 22 nm, a PdI of 0.20 ± 0.02, and a zeta potential of +15.8 ± 0.8 mV. FTIR analysis confirmed electrostatic interactions between CHI and GA. GC–MS identified 24 constituents in the OEO, with carvacrol as the main compound (78.8%). The validated method showed an *R* ^2^ of 0.9976, proving to be specific, precise, and accurate. The encapsulation efficiency was 95% ± 0.7, indicating that the technique preserved the oil’s composition and concentration. The nanocapsules maintained stability under different temperatures with confirmed structural integrity. It is concluded that nanoencapsulation via complex coacervation, combined with experimental design, allows for the production of stable and effective nanocapsules for the delivery of lipophilic bioactive compounds. The validated analytical method reinforces the system’s applicability in future research and the development of functional products.

## Graphical abstract

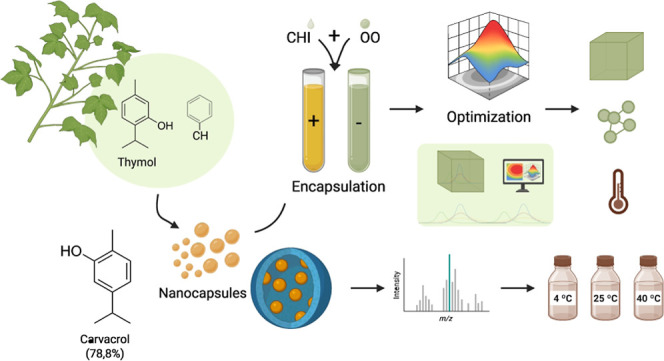

## 1 Introduction

Essential oils are aromatic and hydrophobic compounds extracted from plants. They possess a complex chemical composition, containing secondary metabolites such as terpenoids and phenolic acids, which allow their application in the food, agricultural, and pharmaceutical industries.^1^ Among the vast array of essential oils, oregano essential oil (OEO), extracted from *Origanum vulgare* L., has been extensively utilized as a bioactive compound with antimicrobial, antioxidant, anti-inflammatory, tissue-regenerative, immunomodulatory, and anticancer properties. These activities are primarily attributed to the presence of two monoterpenic phenols—carvacrol (the major component) and thymol—as well as the hydrocarbons *p*-cymene and γ-terpinene.^2^

However, due to the volatile and thermolabile nature of these metabolites, the OEO is susceptible to oxidation, hydrolysis, and loss in industrial processes. To overcome these limitations, the nanoencapsulation of essential oils in polysaccharide-based matrices in the form of nanocapsules has been considered a promising strategy to enhance stability during storage, prolong bioactive properties such as antimicrobial and antioxidant activity, and impart flavor and aroma to food products.^3^

A promising technique for obtaining nanocapsules is complex coacervation, which offers high encapsulation efficiency, controlled release, and improved protection of the encapsulated materials under adverse conditions, such as heat or light exposure. Complex coacervation involves at least two oppositely charged polymers to form the capsule,^4^ typically using polysaccharides and/or proteins when natural biopolymers are desired.^5^

Chitosan (CHI) and gum arabic (GA) are polymers with emulsifying properties and high stability, commonly reported as wall materials for encapsulating a wide range of bioactive compounds.^6^ The primary interaction between CHI and GA occurs through electrostatic attractions, leading to hydrogen bonding, hydrophobic interactions, and physical forces due to their opposite charges.^7^ The nature of these interactions can result in associative or segregative phase behavior, depending on polymer characteristics such as charge density, size, type, and distribution of reactive groups as well as their concentration and ratio. These factors determine the nanoparticle stability and encapsulation efficiency.^8^

To ensure the proper development of the encapsulation method, the resulting nanocapsules must undergo analytical methods capable of identifying and quantifying the encapsulated products in both raw materials and final formulations. Various techniques are available to analyze compound mixtures, phytoextracts, essential oils, and their constituents. Among these, chromatography stands out due to its effectiveness in separating, identifying, and quantifying components in complex product matrices.^9^

In this context, validated analytical methods are necessary to accurately quantify the various compounds present in the OEO-loaded nanocapsules (OEONC). The method suggested for performing qualitative and quantitative analyses of natural and complex compounds, such as nanocapsules containing multiple fractions of different constituents, is gas chromatography coupled with mass spectrometry (GC–MS), especially for detecting carvacrol. High-performance gas chromatographic methods for the separation and quantification of carvacrol in various essential oils have been reported in the literature.^10^ However, there are still no studies reporting the analysis of OEO compounds in complex matrices including biopolymers and nanoparticles. GC–MS, being a simple and efficient technique, may be a suitable analytical tool for the analysis of carvacrol with high reproducibility.

Therefore, this study investigates the influence of the CHI/GA mass ratio on the development of nanocapsules designed for the encapsulation of an OEO. Nanoparticles were prepared via complex coacervation using CHI and GA as natural biopolymers. A Box–Behnken experimental design was employed to optimize the formulation parameters and evaluate their effects on key physicochemical properties of the nanocarriers. Furthermore, the concentration of encapsulated carvacrol, the major bioactive component of the OEO, was quantified using a validated analytical method. The findings contribute to the development of effective biopolymer-based delivery systems for essential oils with potential applications in food preservation and pharmaceuticals.

## 2 Experimental Section

### 2.1 Materials

Oregano essential oil (*Origanum vulgare* L.), batch 84012; density: 0.93 g/mL; refractive index: 1.50 at 20 °C, extracted by steam distillation, was obtained from Ferquima Indústria e Comércio de Óleos Essenciais Ltda. (São Paulo, Brazil). Carvacrol was purchased from Sigma-Aldrich (St. Louis, USA). Gum arabic was acquired from Synth (Diadema, SP, Brazil), and low molecular weight CHI and Tween 80 were obtained from Sigma-Aldrich (St. Quentin Fallavier, France). Acetic acid, sodium hydroxide, and ethyl acetate were supplied by Fisher Scientific (Pittsburgh, PA, USA). Ultrapure water was obtained using a Millipore purification system (Milli-Q Plus, Millipore, St. Quentin en Yvelines, France).

### 2.2 Oregano Essential Oil Composition Analysis

The composition analysis of the OEO was performed by a Gas Chromatograph coupled to a Mass Spectrometer (GC–MS-QP2010 Ultra Shimadzu). Compounds were separated on a capillary RTX-5MS column (30 m × 0.25 mm × 0.25 μm). The injection volume was 1 μL of OEO diluted in ethyl acetate (1:10). The programming of the gas chromatograph oven temperature started at 60 °C, with an increase of 3 °C per min until reaching 240 °C, concluding the run time to be equivalent to 60 min. Injector temperature was maintained at 250 °C, and helium gas was used as the carrier gas at a constant flow rate of 1 mL/min. The mass spectrometer was operated by electron impact with a source temperature of 200 °C, with an ionization energy of 70 V and scan range of *m*/*z* 40 used to create the superlative *m*/*z* 500. For the identification of volatile compounds was used the NIST/EPA/NIH Mass Spectral Database (Version 1.7) own library of GC–MS, confirmed by injection of the carvacrol standard. The quantification of volatile compounds was obtained by normalizing the volatile areas and expressed as a total and percentage of area (%).

### 2.3 Selection of the Optimal Gum Arabic-to-Chitosan (CHI/GA) Mass Ratio

A CHI solution (10 mg/mL) was prepared by dissolving CHI in 1% (v/v) acetic acid under magnetic stirring at 1000 rpm for 8 h at 25 °C. The pH was then adjusted to 5.0 using 1 N NaOH. GA solutions at concentrations of 30, 35, 40, 45, 50, and 60 mg/mL (pH 4.5–5.0) were prepared by dispersing the corresponding amount of gum powder in deionized water under the same stirring conditions. To determine the optimal CHI/GA mass ratio, 1 mL of each GA solution was mixed with 1 mL of the CHI solution. The formulation that resulted in the smallest nanoparticle size and the highest positive surface charge was selected for subsequent experiments.

### 2.4 Nanoparticle Production

The preparation of the nanocapsules followed the methodology adapted from Alvim and Grosso (2010).^11^ 15 mL of the CHI solution was mixed with OEO and Tween 80. The mixture was homogenized using Ultra-Turrax at 8000 rpm for 2 min to form an emulsion. Simultaneously, 15 mL of the GA solution was prepared separately, and the mixture was stirred for 30 min. This solution was then added dropwise to the emulsion under magnetic stirring (600 rpm, 25 °C) and maintained for an additional 60 min. The resulting colloidal dispersion was centrifuged at 4000 rpm for 20 min, and the supernatant was discarded. The pellet was washed three times with ultrapure water (Milli-Q) and subjected to ultrasound treatment for 5 min to ensure a proper dispersion.

The Box–Behnken experimental design was applied to evaluate the influence of three independent concentration variables: OEO (*X*_1_, where −1:0.5%, 0:1.5%, and +1:2.5%), CHI/GA 1:5 ratio (*X*_2_, where −1:2%, 0:3%, and +1:4%), and Tween 80 (*X*_3_, where −1:0.15%, 0:0.3%, and +1:0.45%) on two response variables: particle size (*Y*_1_) and zeta potential (*Y*_2_). The effects and interactions of these variables were analyzed using Statistica software (version 7.0, StatSoft Inc., USA), and response surface plots were generated to visualize their impact on the nanoparticle characteristics.

### 2.5 Nanoparticles Characterization

#### 2.5.1 Size Measurement

Hydrodynamic mean diameter and size distribution of the nanoparticle dispersions were determined at 25 °C by quasi-elastic light scattering using a Zetasizer Nano ZS90 (Malvern Instruments Ltd., Orsay, France). The scattering angle was fixed at 90°. The samples were diluted 1:100 before analysis with Milli-Q water. Each measurement was done in triplicate, meaning that the average was calculated from 9 values.

#### 2.5.2 Zeta Potential Determination

Zeta potential of the nanoparticles was determined by the electrophoretic mobility using Laser Doppler Electrophoresis (Zetasizer Nano ZS90, Malvern Instruments Ltd., Orsay, France). Nanoparticle dispersions were diluted (1:100) with NaCl at 1 mmol/L. Values are presented as means of measurements performed on three replicate samples.

#### 2.5.3 Scanning Electron Microscopy (SEM)

The morphology of the freeze-dried nanocapsules was examined to study the surface structures of powders by scanning electron microscopy (SEM; Leo EVO-40 VPX, Carl Zeiss SMT, Cambridge, UK). The samples were added to adhesive tape mounted on the specimen stubs, and particles were covered with gold–palladium before analysis.

#### 2.5.4 Atomic Force Microscopy

Atomic Force Microscopy (AFM) micrographs were obtained under a microscope SPM-9700 (Shimadzu, Japan), operating in intermittent contact mode at room temperature. Samples containing 0.002% (w/w) in ultrapure water were spread on a mica surface and dried at room temperature for 48 h. Silicon cantilevers (Pointprobe, NanoWorld AG, Switzerland) were used to obtain the images by using a spring constant in the range of 21–78 N/m. The resonance frequency was set at 320 kHz, and the scanning rate was 1 Hz. An area of 5 μm × 5 μm was analyzed for each sample.

#### 2.5.5 Fourier Transform Infrared Spectrometry

The CHI, GA, and OEONC were evaluated by Fourier Transform Infrared Spectrometry (FTIR). The samples were prepared on KBr disks, and the transmittance was measured from 500 to 4000 cm^–1^ using an FT/IR-4600 instrument (Jasco, Maryland, EUA). The data were processed (Origin 8, OriginLab Northampton, USA), and the final graphs were plotted.

#### 2.5.6 Thermal Analysis

Thermogravimetric (TG) and Differential Scanning Calorimetry (DSC) curves were obtained using simultaneous thermal analysis equipment (PerkinElmer Pyris 6). Samples of around 5 mg were placed in sealed aluminum crucibles with pierced lids. Measurements were taken from room temperature up to 400 °C using a heating rate of 10 °C/min. The sensors and crucibles were immersed in a constant flow of nitrogen (70 mL/min) during the experiment.

### 2.6 Temperature Stability

To assess the temperature stability, the nanocapsules were conditioned in glass vials at three different temperatures, under refrigeration at 4 °C with a relative humidity of 53%, at room temperature 25 °C, and in an oven at 40 °C.^12^ The thermal stability test was carried out for 120 days. The nanoparticles’ size and zeta potential were evaluated after 7, 15, 30, 60, 90, and 120 days. Three batches of nanosystems were produced, and each batch was fractionated to be subjected to each temperature evaluated. All assays were performed in triplicate.

### 2.7 Validation Methods

The chromatographic method validation was performed according to the International Conference on Harmonization (ICH) guidelines,^13^ assessing specificity/selectivity, linearity, precision, accuracy, robustness, and the limits of detection (LOD) and quantification (LOQ). Validation tests followed Good Manufacturing Practices (GMP), and all volumetric equipment was evaluated and calibrated before analysis. The analytical balance (Sartorius Analytical Balance MSA-224S-000-DU Cubis, Elk Grove, USA) was calibrated to a minimum of 1 mg. Three individual stock solutions of 10 mg of carvacrol were prepared in ethyl acetate, transferred into airtight vials, and stored at −20 °C until use. Working solutions with concentrations ranging from 5 to 25 μg/mL (5, 10, 15, 20, and 25 μg/mL) were prepared and used for the validation analyses.

#### 2.7.1 Specificity/Selectivity

The selectivity of the analytical method was assessed by injecting solutions containing 100% of the normal working concentrations of carvacrol, oregano oil, and nanocapsules. These analyses were repeated six times. The ability to separate all compounds and demonstrate no chromatographic interference from standard samples was thoroughly analyzed.

#### 2.7.2 Linearity

Calibration curves for carvacrol were prepared by injecting standard solutions ranging from 5 to 25 μg/mL (5, 10, 15, 20, and 25 μg/mL). The peak area of the standards was plotted against the analyte concentrations. Analytical curves were developed by calculating the regression line using the least-squares method. This experiment was conducted in triplicate on different days.

#### 2.7.3 Limit of Detection and Limit of Quantitation

The limit of detection (LOD) was determined based on the ratio of the standard deviation of the response to the estimated slope from the standard calibration curve, multiplied by 3. The limit of quantitation (LOQ) was defined as the smallest amount of analyte that could be reproducibly quantified. This parameter was calculated by the ratio of the standard deviation of the response to the slope of the standard calibration curve multiplied by 10.

#### 2.7.4 Accuracy

Accuracy was determined through six repetitions of carvacrol concentrations (ranging from 5, 15, and 25 μg/mL) in 1 mL of ethyl acetate. The found concentrations were obtained by substituting the peak response ratios of the carvacrol solutions at the added concentrations into a derived regression equation. The found and added concentrations were then used to determine the absolute percentage deviation at each carvacrol concentration contained in oregano oil.

#### 2.7.5 Precision

The precision of the method was evaluated by analyzing the intraday and interday variability of carvacrol samples. Intraday precision was determined by quantifying carvacrol samples at three different concentrations (5, 15, and 25 μg/mL). The samples were injected six times and prepared on the same day. Interday precision was assessed separately from the peak areas obtained by injecting the same three concentrations of the phytoconstituent on different days. Both interday and intraday precision were expressed as the relative standard deviation (% RSD).

#### 2.7.6 Encapsulation Efficiency

Nanocapsules were diluted in 1 mL of ethyl acetate, sonicated for 15 min, and then centrifuged (Eppendorf centrifuge 5418, Rotor FA-45-18-11, Hamburg, Germany) at 15,000 rpm for 20 min to remove any potential precipitate. Samples were subsequently diluted in ethyl acetate and filtered through a 0.22 μm Millipore filter before GC–MS analysis. The essential oil content within the nanocapsules was determined using the carvacrol standard curve and a validated method developed previously.

### 2.8 Statistical Analysis

All experiments were performed in triplicate, and the results are expressed as mean ± standard deviation. Statistical significance was assessed using one-way analysis of variance (ANOVA) followed by Tukey’s post hoc test for multiple comparisons. Differences were considered statistically significant at a confidence level of *p* < 0.05. All analyses were carried out using GraphPad Prism 9.0.

## 3 Results and Discussion

### 3.1 Compositional Analysis of OEO by Gas Chromatography–Mass Spectrometry

A total of 24 constituents were identified in the OEO used in this study. The constituents detected in amounts greater than 0.3% are reported in Table 1. Carvacrol (78.8%), *p*-cymene (10.4%), γ-terpinene (5.7%), linalool (1.5%), and thymol (1.2%) were detected as the major compounds. Other constituents, such as α-pinene (0.5%), myrcene (0.3%), β-caryophyllene (0.7%), and thymol methyl ether (0.5%), were detected in smaller amounts. Previous studies, in the literature, have also reported carvacrol and *p*-cymene as predominant constituents in OEO.^14,15^ The quantities of the main constituents detected in OEO can vary due to environmental conditions (for example, altitude, temperature, precipitation, and geographic distribution) at the source of the plant.^16^ Thus, knowledge of the chemical composition can help to understand similarities or differences in the biological activities observed in different essential oils of the same plant species.

**Table 1:** Compounds Identified in the Essential Oil of *Origanum vulgare* L.^a^

peak	retention time	compound	concentration (%)
**1**	3.779	α-pinene	0.46
**2**	4.522	myrcene	0.35
**3**	5.241	*p*-cymene	10.45
**4**	5.917	γ-terpinene	5.69
**5**	6.772	linalool	1.47
**6**	10.775	thymol methyl ether	0.50
**7**	12.265	thymol	1.23
**8**	12.793	carvacrol	78.82
**9**	16.384	β-caryophyllene	0.73
		total detected	99.70

a Results expressed as a percentage (%) of the total area for constituents with a percentage area > 0.3%.

### 3.2 Nanoparticle Production

#### 3.2.1 Interactions between the Biopolymers and Nanoparticle Preparation

The nanocapsules were prepared using the complex coacervation technique, which involves two main steps: (1) emulsification, which determines the size and distribution of the nanocapsules, and (2) the formation of the polymeric shell surrounding the droplets. The emulsification step can be influenced by several physical parameters, including equipment configuration, stirring rate, volumetric ratio between the phases, and the physicochemical properties of the components, such as interfacial tension, viscosity, density, and chemical composition.^17^ The second step is governed by the ability of the two biopolymers to form complexes through electrostatic interactions, due to their opposite charges.^18^ The efficiency of this complexation depends on the charge density of both polymers, which is influenced by factors such as pH, ionic strength, total polymer concentration, and the mass ratio between the polymers.^19,20^

Each stage of the process was evaluated by analyzing the influence of the polymeric components on the particle size, surface charge, and mechanism of nanocapsule formation. To determine the optimal conditions for synthesizing oregano oil-loaded CHI/GA (CHI/GA) nanocapsules (OEONC), the study focused on identifying the ideal CHI/GA ratio to achieve efficient coacervation. This relationship is illustrated in Figure 1, which shows the effect of different polymer ratios on particle size and zeta potential.

**Figure 1:**
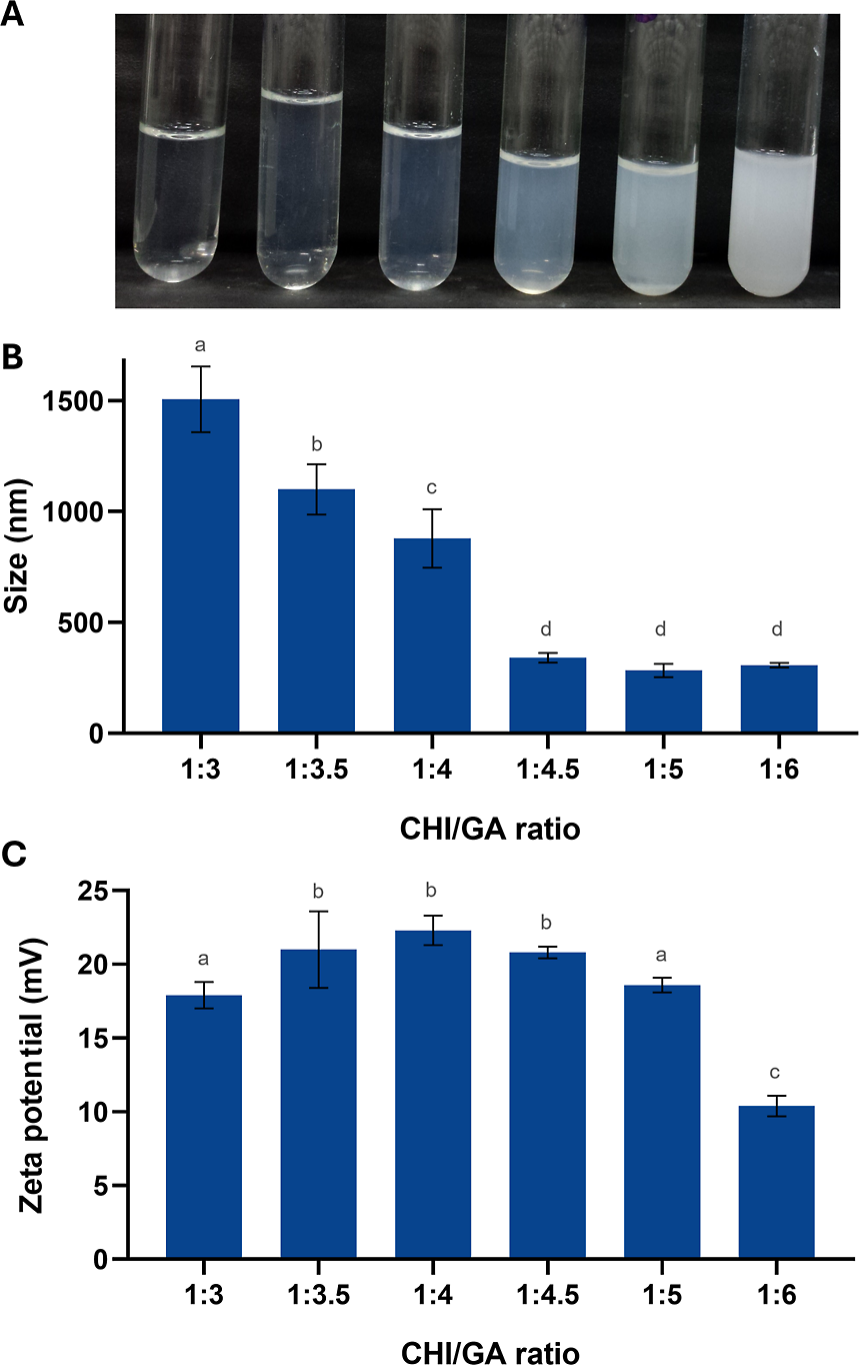
CHI/GA ratio for the production of nanoparticles. (A) Macroscopic aspect of the polymer ratio, (B) particle size, and (C) zeta potential of CHI solution (10 mg/mL) with increasing concentrations of GA (30 to 60 mg/mL). Different superscript letters indicate statistically significant differences between groups (*p* < 0.05).

The formulations exhibited slight variations in particle size; however, the CHI/GA ratios of 1:3 and 1:4 resulted in significantly larger average sizes, exceeding the nanometer scale (Figure 1B). When the GA concentration was increased, a single, sharp peak in particle size distribution was observed, indicating uniform dispersion with a low polydispersity index. Zeta potential values remained positive, consistently above +10 mV, regardless of the GA concentration (Figure 1C). Nevertheless, at the highest GA ratio tested (CHI/GA 1:6), a reduction in zeta potential was observed, attributed to the excess negative charge contributed by the anionic polysaccharide.

These findings confirm that electrostatic interactions are the main driving force for the formation of small, homogeneously dispersed CHI/GA nanoparticles. Furthermore, the positive charges from CHI are predominantly located, which suggests that this polymer is located on the surface of the nanoparticle shell, as illustrated in Figure 2.

**Figure 2:**
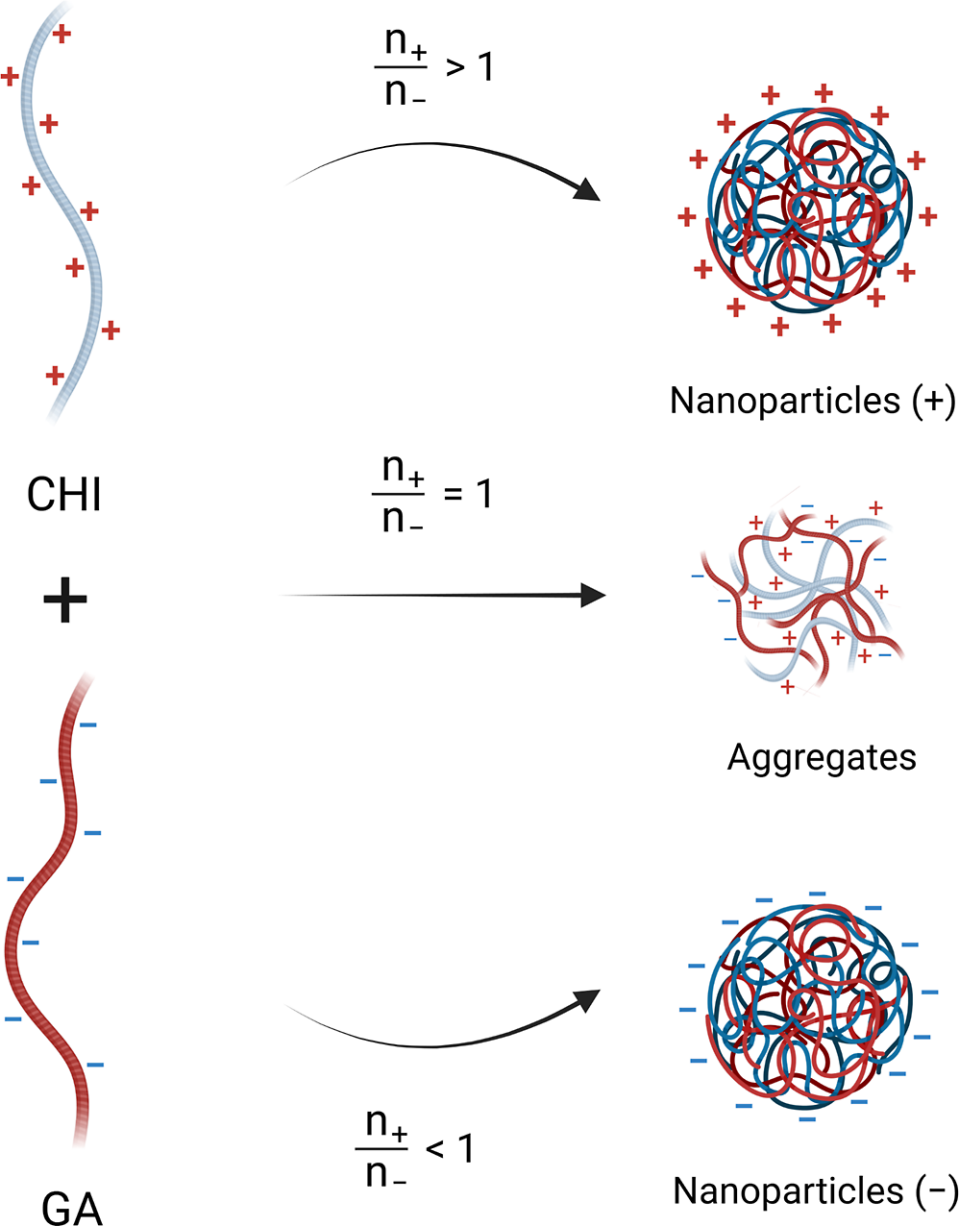
Effect of the polyelectrolyte charge ratio on the particle size and surface charge of the colloidal complexes.

GA contains numerous carboxylic acid groups in its main monomer unit (β-d-galactopyranose, substituted at position 6 with α-1-arabinofuranose side chains), which exhibits a p*K*_a_ of approximately 3.5 and develops negative charges across a broad pH range.^21^ In contrast, CHI contains glucosamine units, whose amine groups have a p*K*_a_ of around 6.8. Unlike most polysaccharides, CHI becomes positively charged over a wide pH range, especially under acidic conditions.^22^ Previous studies have shown that the interaction between low molecular weight CHI and GA leads to electrostatic complexation, resulting in a milky colloidal dispersion.^23,24^ Once both polyelectrolytes interact, multiple electrostatic interactions occur along the CHI chains, leading to the formation of a stable polyelectrolyte complex.^25^

Based on these results, the CHI/GA ratio of 1:5 was selected as optimal, as it yielded nanoparticles with the smallest average diameter (ranging from 290 to 315 nm) and the highest positive surface charge (+15.89 ± 4 mV). These findings align with those reported by Astutiningsih et al. (2022),^26^ who found that a 1:5 core-to-wall ratio using CHI and GA was optimal for forming stable saffron essential oil nanoparticles with desirable physicochemical properties. In contrast, Rajabi et al. (2019)^27^ found a 2:1 CHI/GA ratio more suitable for encapsulating saffron extract. These variations suggest that the ideal biopolymer ratio depends on several factors, including the preparation method, core material type and concentration, wall material ratio, molecular weight, and size of the molecules. All of these parameters directly influence the final particle size and zeta potential of the nanocapsules.

#### 3.2.2 Box–Behnken Design Optimization of Nanocapsules

Based on the results from the initial nanoparticle preparation phase, the synthesis conditions for CHI/GA nanocapsules containing an OEO were optimized. An experimental design approach was applied to minimize the number of required experiments, thereby reducing the time and material consumption. Optimization was conducted using a Box–Behnken Design (BBD), a second-order model based on an incomplete three-level factorial design. Three independent variables were evaluated: OEO concentration (*X*_1_), polymer concentration (CHI/GA at a 1:5 ratio) (*X*_2_), and nonionic surfactant concentration (Tween 80) (*X*_3_). The optimal parameters were those that resulted in the smallest particle size and the highest positive surface charge.

The Pareto chart (Figure 3) presents the relative significance of the main effects and interaction terms of the independent variables (OEO, CHI/GA, and Tween 80) on the dependent responses (particle size and zeta potential). This analysis identified statistically significant variables (*p* < 0.05). The length of each bar represents the standardized effect of each variable. Negative values indicate an antagonistic effect—unfavorable for achieving the desired characteristics of small size and high positive zeta potential—while positive values suggest a favorable or synergistic effect.^28^

**Figure 3:**
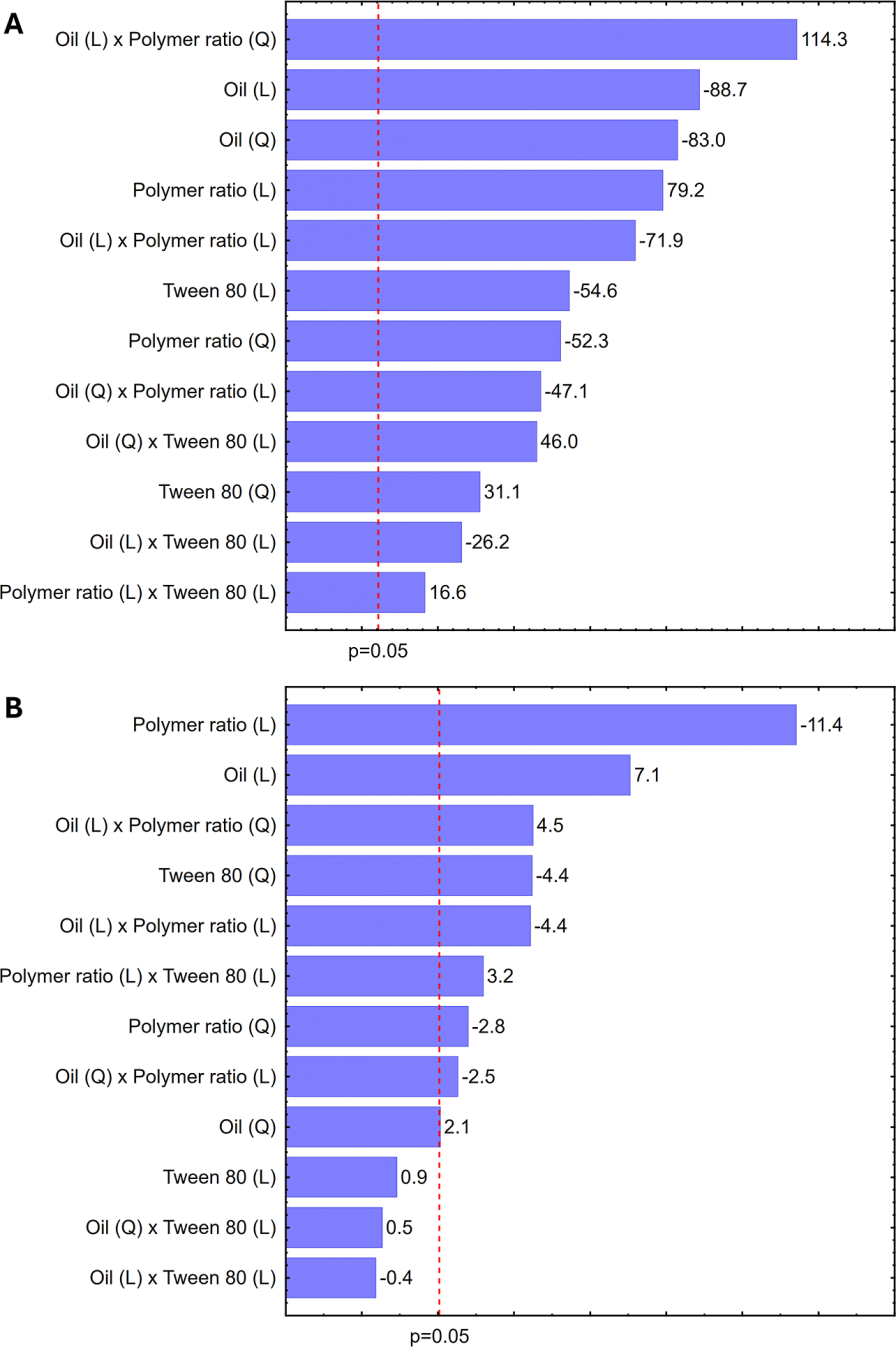
Pareto chart illustrating the effects of formulation variables on (A) particle size and (B) zeta potential.

All variables significantly influenced particle size (*R* ^2^ = 0.999, *R* ^2^_adj_ = 0.999). Except for the surfactant concentration, the independent variables also had a significant impact on the zeta potential (*R* ^2^ = 0.913, *R* ^2^_adj_ = 0.881).

The interaction between the CHI/GA polymer ratio and oregano oil exerted the greatest positive influence on particle size, followed by that of the oil alone. Thus, larger nanocapsules were primarily associated with higher concentrations of polymer, while the OEO tended to generate smaller particles. For the zeta potential, the OEO was also the variable responsible for the highest zeta potential values. These higher values suggest good colloidal stability, as a higher absolute zeta potential (positive or negative) increases repulsive forces between particles. Thus, the zeta potential, whether negative or positive, significantly influences colloidal stability, confirming that particle charge magnitude, rather than its direction, plays a central role in preventing aggregation and enhancing system stability.

To evaluate the direct effects of experimental variables, three-dimensional response surface plots were constructed. Figure 4A–C presents the response surface plots for particle size, which ranged from 161 to 5528 nm. The darker green region in the plots corresponds to the smallest nanoparticle sizes. The surfaces indicate that lower polymer concentrations, combined with higher amounts of the OEO and surfactant, yield smaller nanocapsules, especially with higher surfactant concentration. The response for particle size varied linearly with the tested variables, confirming significant interactions between all of the independent factors and the final size of the nanocapsules under different conditions.

**Figure 4:**
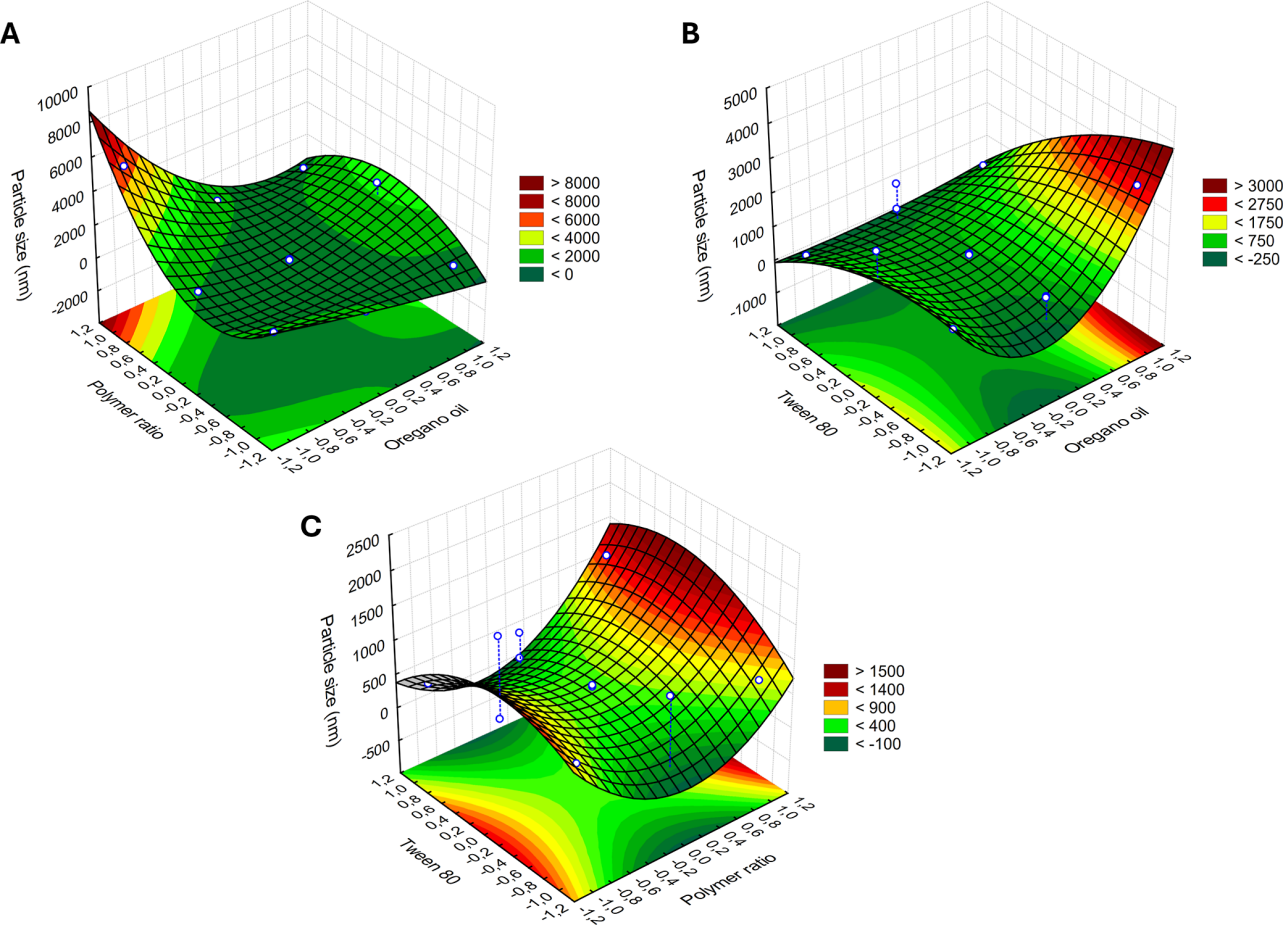
Response surface plots showing the effects of oregano oil (*X*_1_), CHI/GA polymer ratio (*X*_2_), and Tween 80 (*X*_3_) on nanocapsule particle size.

The oregano oil-loaded nanocapsules exhibited zeta potential values ranging from −2.83 to +19.7 ± 0.8 mV. Figure 5 presents the response surface plot related to the zeta potential of the nanoparticles. The concentration of the nonionic surfactant Tween 80 did not significantly affect the zeta potential values. The region of interest in the plot appears dark red, corresponding to the highest positive zeta potential values observed. The surface response indicates that increasing the OEO while decreasing the polymer concentration led to higher zeta potentials.

**Figure 5:**
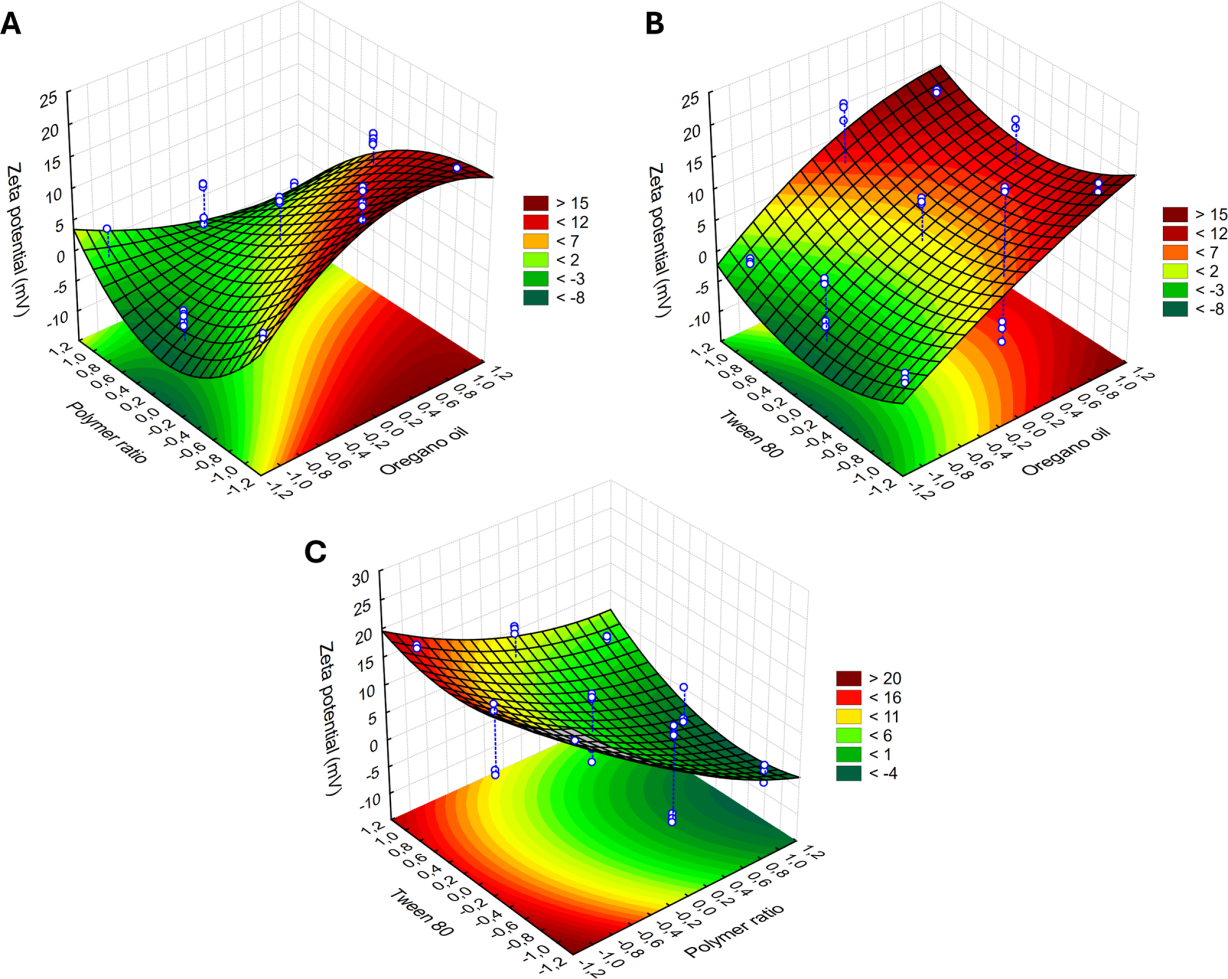
Response surface plot showing the influence of OEO, polymer ratio (CHI/GA), and surfactant concentration on the zeta potential of nanocapsules.

For instance, Abdel-Hakeem et al. (2022)^29^ developed CHI nanoparticles cross-linked with TPP and coloaded with gentamicin and ascorbic acid. The resulting nanocarriers exhibited a zeta potential of +30.01 mV and significantly enhanced antimicrobial activity while also minimizing cytotoxicity. Similarly, El-Naggar et al. (2023)^30^ obtained CHI nanoparticles using a comparable method and reported zeta potentials of +33.1 mV, attributed to the presence of protonated amine groups on the nanoparticle surface.

The Box–Behnken Design optimization study successfully identified the ideal formulation conditions to obtain nanocapsules with the desired physicochemical properties. The optimized formulation consisted of 470 mg of OEO, 659 mg of CHI/GA (1:5), and 13 mg of Tween 80. This formulation yielded nanoparticles with a mean particle size of 323 ± 22 nm, a unimodal size distribution with a polydispersity index (PdI) of 0.20 ± 0.02, and a zeta potential of +15.8 ± 0.8 mV.

#### 3.2.3 Morphological Analyses of OEONC

The Scanning Electron Microscopy (SEM) images of the nanocapsules are shown in Figure 6A. They provide useful information about the particle size and morphology. The nanocapsules, as expected, are spherical and well-individualized, with typical images of nanocapsules composed of only a core surrounded by a polymer shell, whose average size was found below 300 nm. Sonar et al. (2023)^31^ obtained CHI and OEO nanoparticles with similar morphology and size, ranging from 100 to 200 nm.

**Figure 6:**
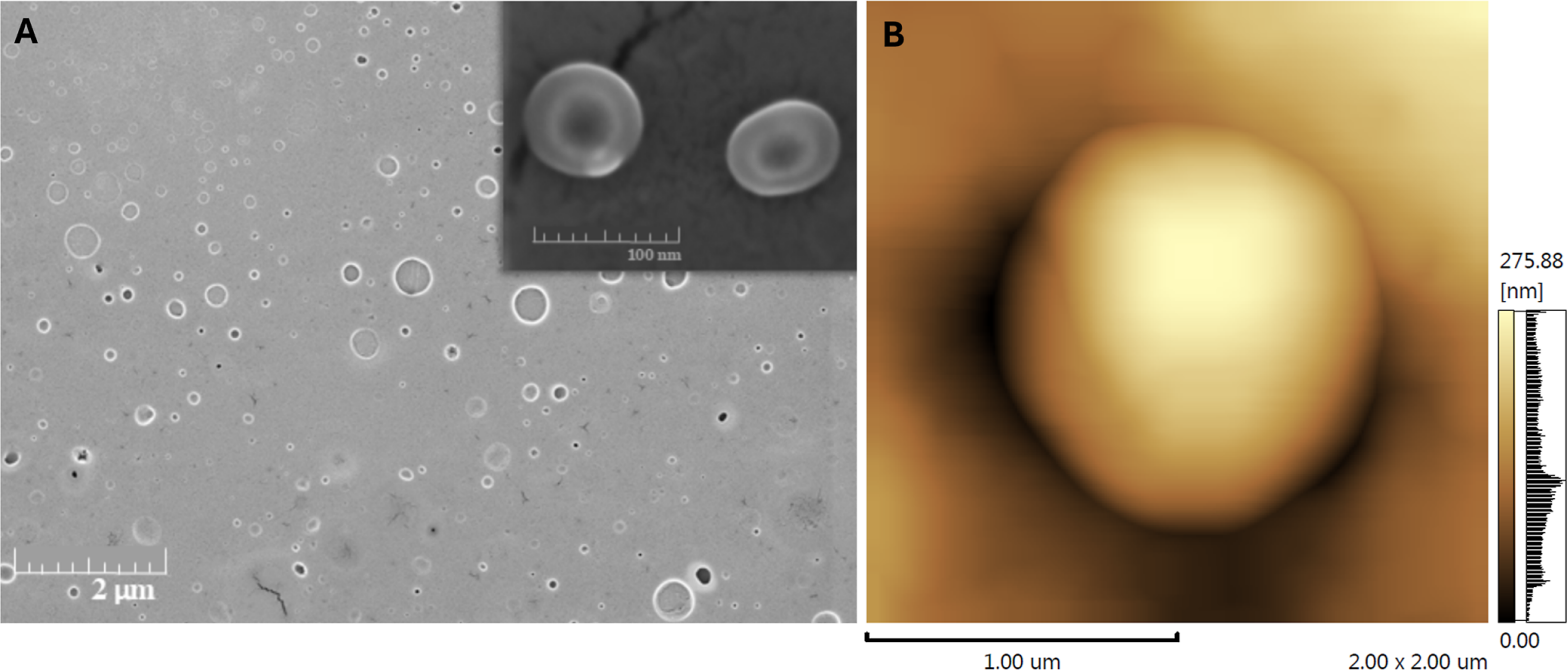
Scanning electron microscopy images (×80k (A) and ×30k (B)) showing the morphology of OEO-loaded CHI/GA nanocapsules (**A**) and AFM measurements of oregano oil-loaded nanocapsules produced using GA and CHI polymers (**B**).

AFM imaging (Figure 6B) revealed a three-dimensional spherical shape with a smooth surface. This morphological pattern is consistent with that previously reported for CHI–carrageenan nanosystems.^32^ Furthermore, the average particle size measured by AFM was 275 ± 0.8 nm, which corroborates the values obtained by the other techniques described.

#### 3.2.4 Fourier Transform Infrared Spectrometry

The FTIR spectrum of GA (Figure 7) showed characteristic bands between 1600 and 750 cm^–1^, attributed to the asymmetric and symmetric stretching vibrations of the carboxylate group (–COO^–^), in addition to bands associated with the C–O–C vibrations of the glycosidic bond. Although the broad stretching band of the hydroxyl group (–OH) normally occurs in the region of 3200 to 3600 cm^–1^, the analyzed range also includes deformations related to this group.^33,34^ The FTIR spectrum of CHI exhibited characteristic peaks as follows: the band around 1630–1650 cm^–1^ corresponds to the C=O stretching vibration of the amide group (amide I band); the peak near 1500 cm^–1^ is attributed to a combination of N–H bending and C–N stretching vibrations (amide II); the band at approximately 1375 cm^–1^ is associated with –CH_3_ symmetric deformation in the amide group; the absorption near 1300 cm^–1^ is related to –CH_2_ stretching vibration from the pyranose ring. Additionally, specific bands observed in the 1050–1130 cm^–1^ range correspond to C–O and C–O–C stretching vibrations typical of the β (1 → 4) linked glycosidic bonds, confirming the polysaccharide structure of CHI.^35,36^ A slightly different trend was observed in the FTIR spectra of the nanocapsules. As a result of the interactions between the biopolymers, there was a change in a region corresponding to the carbonyl amide group, with the bending vibration of the N–H group being shifted to approximately 1250 cm^–1^. This shift can be attributed to electrostatic interactions between the negative functional groups of GA and the positive groups of CHI, responsible for the coacervation phenomenon.^24,37^

**Figure 7:**
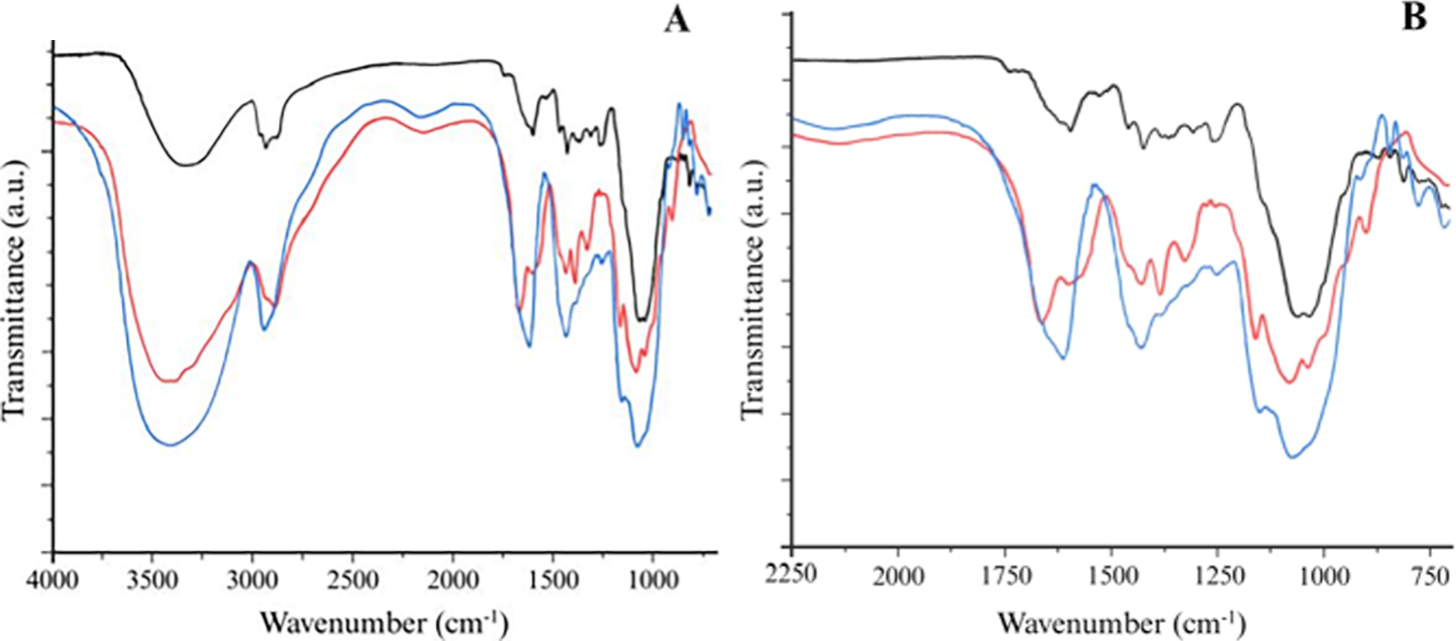
Fourier Transform Infrared (FTIR) spectra of OEONC (black), GA (blue), and CHI (red) samples.

#### 3.2.5 Thermal Analysis

Thermal stability is a fundamental parameter in evaluating the performance of nanoparticles as encapsulation and storage materials. Thus, the thermal stability of GA, CHI, and OEONC was analyzed by thermogravimetry (TGA) (Figure 8A). GA and CHI exhibited a single main mass loss step, with decomposition peaks around 280 °C, mainly attributed to the degradation and evaporation of the macromolecular chains, resulting in similar characteristics to those presented by Cavalcanti et al. (2021).^38^ For nanoparticles, mass loss started around 250 °C, and practically no residue was observed at 400 °C, suggesting complete volatilization of the lipophilic core, probably associated with the decomposition of the encapsulated essential oil.^12,39^ In the CHI curve, even at high temperatures, the mass was preserved, indicating a greater thermal stability of the isolated polymer. Furthermore, the nanoparticles presented a second mass loss stage close to 450 °C, indicating the occurrence of thermal degradation and carbonization of the lipid matrix, as well as the fact that the biopolymers used as a matrix were able to efficiently encapsulate the oregano oil, giving it greater thermal resistance during processing.^40^

**Figure 8:**
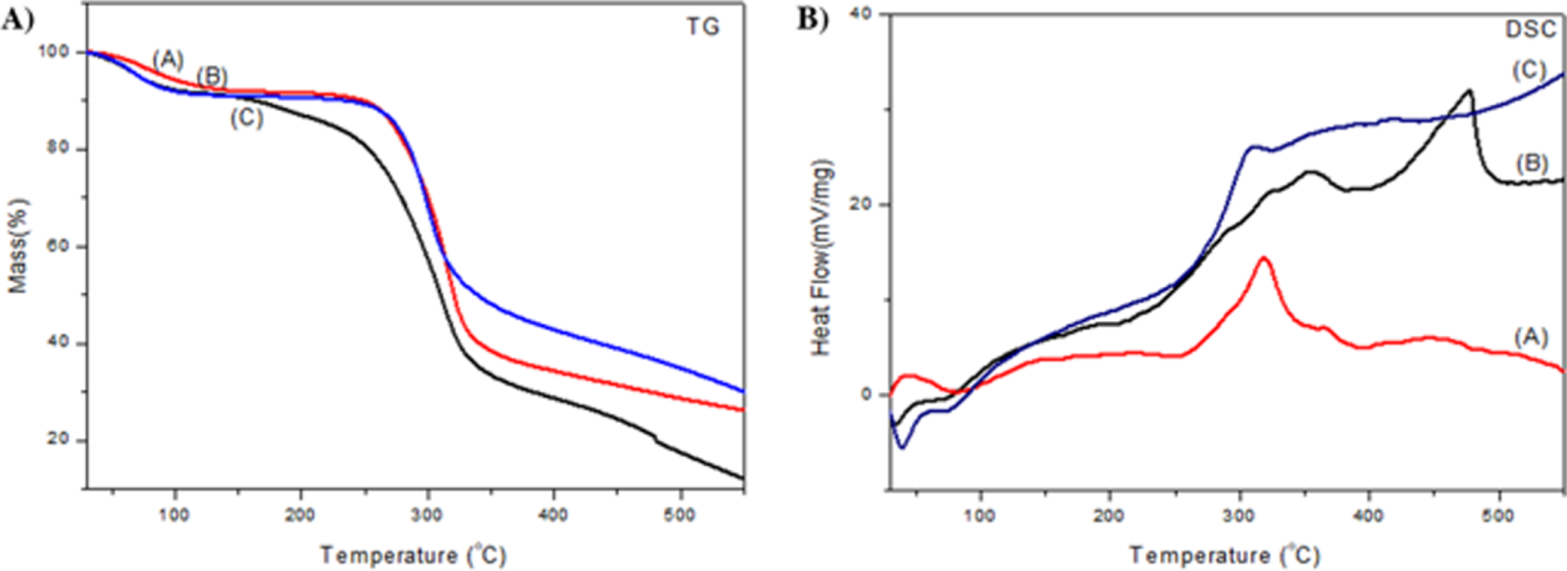
Thermogravimetric (TG) and Differential Scanning Calorimetry (DSC) of the samples: (A) GA, (B) OEONC, and (C) CHI.

Complementary analysis by differential scanning calorimetry (DSC), also represented in Figure 8B, revealed that the melting point of CHI and nanoparticles begins after 50 °C, with an endothermic peak at around 48 °C. In contrast, GA presented a discrete exothermic peak, probably related to crystallization. Above 100 °C, a broad exothermic trend was observed, with peaks close to 250 °C. The formulation containing oregano oil presented a single sharp endothermic peak at 400 °C, indicating oxidative degradation of the volatile compounds. This behavior was not observed in the isolated spectra of GA and CHI, which suggests the presence of compounds in crystalline form and possible physical interaction between the components of the polymer matrix and the oily core.

### 3.3 Temperature Stability of OEONC

The stability of the OEONC was evaluated in terms of the particle size, polydispersity index (PdI), and zeta potential over 120 days of storage at 4 °C, 25 °C, and 40 °C (Figure 9). The stability of the nanoparticles was significantly affected by both storage temperature and time, as shown by two-way ANOVA with Tukey’s post hoc test (*p* < 0.05). Particle size (Figure 9A) increased significantly over 120 days under all storage conditions, with samples at 40 °C showing the largest diameters compared to those at 25 and 4 °C. Differences between 25 and 4 °C became significant only after 60 days (*p* < 0.01), with 4 °C samples exhibiting the smallest size increase. These slight increases are likely due to self-aggregation of the polymeric components and do not substantially impact system performance.^41^

**Figure 9:**
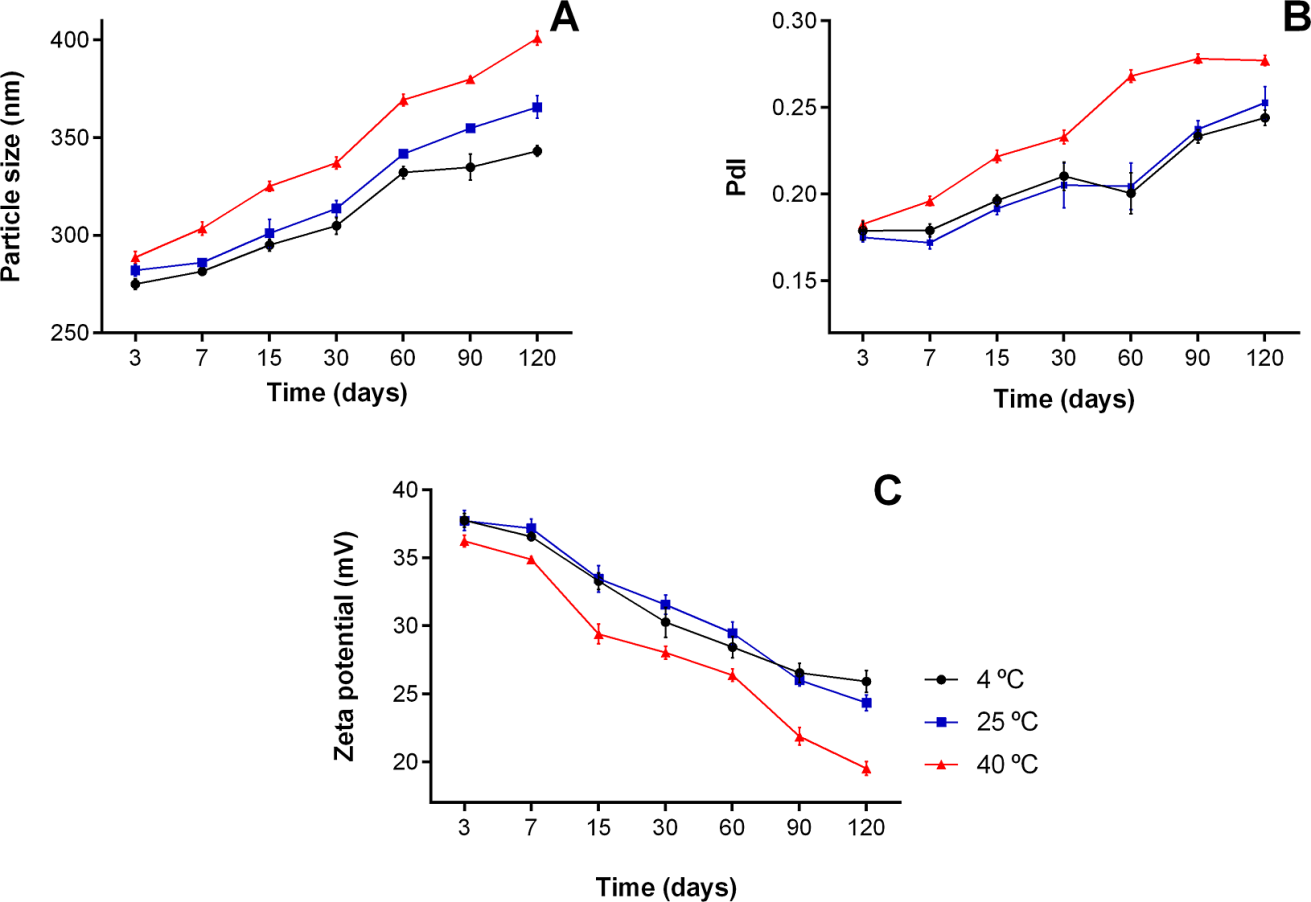
Particle size (**A**), PdI (**B**), and zeta potential (**C**) of nanocapsules stored at 4 °C, 25 °C, and 40 °C for 120 days.

The PdI value increased with time (Figure 9B), although differences between 4 and 25 °C were not statistically significant (*p* > 0.05). Overall, PdI remained below 0.3, indicating excellent homogeneity and monodispersity of the nanoformulations.^12^ Nanocapsules stored at 40 °C showed higher PdI values from day 30, reaching significance relative to the other groups (*p* < 0.05), indicating reduced homogeneity. These results suggest that elevated temperature promotes particle population broadening, while lower temperatures preserve distribution uniformity.

Regarding the zeta potential, a slight decrease in positive values over time under all storage conditions was also observed (Figure 9C). The reduction was significantly more pronounced at 40 °C, while no significant differences were detected between 4 and 25 °C (*p* > 0.05). This reduction is attributed to the partial neutralization or rearrangement of the positive charges of CHI, which contributes to the electrostatic stabilization of the nanoparticles. Despite the gradual decline, values remained above +25 mV at 4 and 25 °C, consistent with preserved colloidal stability.

These findings are consistent with those reported by Hussein and collaborators (2020),^42^ who observed comparable stability in lipid nanocapsules containing orange and eucalyptus essential oils. Therefore, the ability of nanocapsules to retain essential oils under different storage conditions and durations is a key indicator of the stability of the system.^41^ Overall, the results indicate that OEONC formulations, particularly those stored at 4 and 25 °C, maintain stability and effectively retain the encapsulated essential oil, confirming the system’s thermal and colloidal stability.

### 3.4 Validation Methods for Dosing OEO into Nanocapsules

Studies concerning the composition of essential oils and their constituents have revealed a significant diversity of volatile substances within these samples, making the separation and identification of compounds of interest challenging. Gas chromatography has emerged as a crucial tool due to its practicality and high efficiency in separating complex samples, especially when coupled with detectors like the mass spectrometer.^43^ Selectivity is evaluated to ensure that the analyte’s response peak (assessed at its characteristic retention time) originates exclusively from the analyte itself and not from other interfering compounds present in the sample.

Chromatograms of carvacrol, oregano oil, and the nanocapsules showed no potential interferences in the quantification of carvacrol contained in the OEO (Figure 10). This was because the compounds did not exhibit a mass-to-charge ratio identical to that of carvacrol, demonstrating the selectivity of the quantification method.^45^

**Figure 10:**
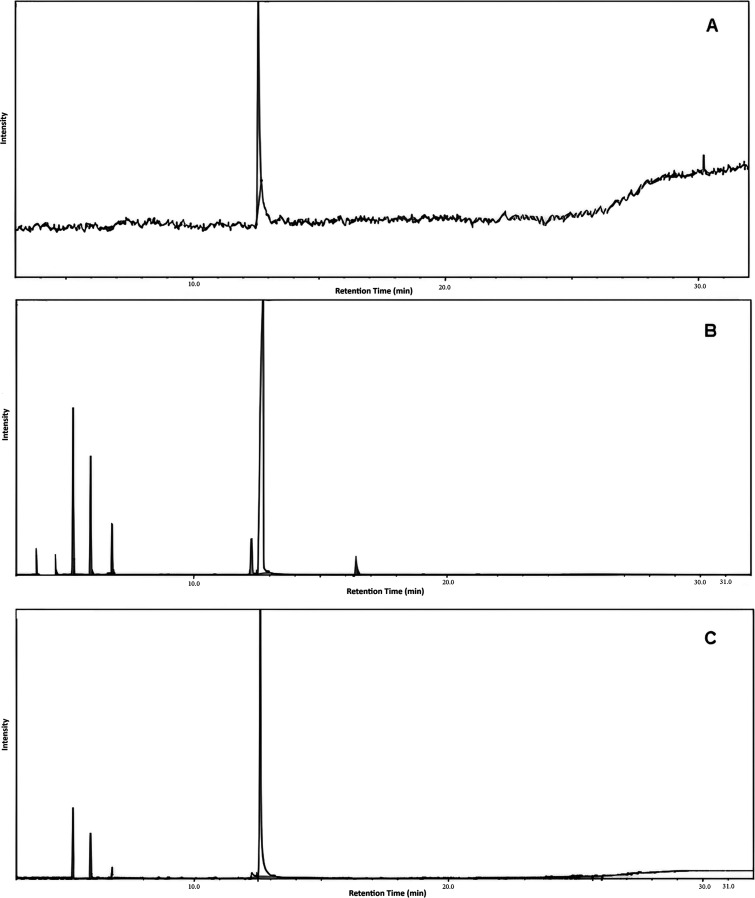
Representative GC/MS chromatograms of the (A) carvacrol, (B) oregano oil, and (C) nanocapsules containing OEO.

Linearity was expressed as the correlation coefficient of the analytical curve. The equation of the line, determined using the least-squares method, was *y* = 21.346*x* + 9.5120, where *x* represents the carvacrol concentration in μg/mL and *y* represents the area, both obtained by GC–MS. The method exhibited a correlation coefficient (*R* ^2^) of 0.9976. This coefficient is a parameter that allows for the estimation of the quality of the obtained curve as values greater than 0.99 indicate the presence of a linear response within the studied concentration range. Thus, the results demonstrate that the analytical curve can be effectively used to quantify samples containing carvacrol, confirming the validity of the method, according to linearity.

The method demonstrated an LOD of 0.22 μg/mL and an LOQ of 0.66 μg/mL, indicating satisfactory limits. These values were lower than the lowest concentration used in the construction of the linear curve (5 μg/mL). Although GC–MS is widely employed for quantifying carvacrol in essential oils, there are no specific studies detailing the LOD and LOQ of this compound in oregano oil. This parameter is crucial for validating and interpreting results, as it defines the sensitivity and quantitative capability of the method.^46^

The accuracy of carvacrol was found to be between 99% and 102%, with an RSD value of 3.59%. Precision was estimated by both intraday (repeatability) and interday precision. The intraday and interday relative standard deviations (RSD) were less than or equal to 1.06% and 1.22%, respectively. These low values indicate that the method exhibited minimal random errors (*P* < 0.05), proving efficient for the determination of carvacrol in nanocapsules (Table 2).

**Table 2:** Intra- and Interday Precision of Carvacrol by GC/MS^a^

	carvacrol measured concentration		
concentration (μg/mL)	mean (μg/mL)	SD	RSD (%)
intraday variation			
5	5	0.05	1.01
10	15.2	0.16	1.06
25	24.7	0.26	1.04
interday variation			
5	5	0.05	0.95
10	15.5	0.18	1.22
25	24.7	0.26	1.06

a Values are for *n* = 3 observations.

The results indicated that the nanoencapsulation process completely preserved the oil composition and concentration of each one, achieving an encapsulation efficiency of high oregano oil of 95% ± 0.7. The high encapsulation efficiency of the OEO indicates that in the nanocapsule production process, the oil immediately diffused into the core, where the polymeric envelope surrounded it. Thus, there was a small amount of essential oil that was lost during the production of the nanocapsules. High EE% values are a desirable feature in the encapsulation technique because they increase the shelf life of the oil and ensure the stability of the nanosystem.^47^

Taken together, beyond the scientific contribution to understanding the interactions between natural biopolymers and lipophilic compounds, the results obtained in this study hold significant potential for diverse industrial applications. In the food sector, these nanocapsules may serve as effective carriers for preserving and gradually releasing antioxidant and antimicrobial compounds, contributing to the replacement of synthetic additives in functional foods and active packaging. In pharmaceuticals, the system shows promise for oral or topical formulations aimed at treating infectious and inflammatory diseases as well as serving as an adjuvant in anticancer therapies. In agriculture, their application as controlled-release biofungicides or bioinsecticides represents a sustainable alternative to conventional chemical inputs. Therefore, future development of multifunctional products could expand their impact across health, nutrition, and environmental sustainability, providing a clear pathway for translating these findings into clinically and commercially relevant solutions.

## 4 Conclusion

In this work, we report the successful development of CHI/GA nanocapsules encapsulating an OEO through complex coacervation, guided by a Box–Behnken experimental design. The optimized formulation achieved a favorable particle size, charge distribution, and structural integrity, which were confirmed by comprehensive physicochemical characterizations. The nanocapsules also exhibited robust stability under varying storage conditions, underscoring their potential as a promising delivery system for lipophilic bioactives. Notably, the validated GC–MS method demonstrated strong specificity and sensitivity for quantifying carvacrol within complex polymeric matrices, enabling the determination of high encapsulation efficiency and filling a methodological gap in the literature. Future research could explore in vivo evaluation of pharmacokinetics, efficacy, and safety, long-term stability studies, production scale-up, and incorporation of other bioactive compounds to expand formulation utility. Overall, these findings in this work not only reinforce the versatility of natural biopolymers for encapsulation strategies but also offer a scalable and analytically validated approach for enhancing the functionality of the OEO.

## Data Availability

Data will be made available on request.
